# Adenovirus-vectored novel African Swine Fever Virus antigens elicit robust immune responses in swine

**DOI:** 10.1371/journal.pone.0177007

**Published:** 2017-05-08

**Authors:** Shehnaz Lokhandwala, Suryakant D. Waghela, Jocelyn Bray, Neha Sangewar, Chloe Charendoff, Cameron L. Martin, Wisam S. Hassan, Tsvetoslav Koynarski, Lindsay Gabbert, Thomas G. Burrage, David Brake, John Neilan, Waithaka Mwangi

**Affiliations:** 1 Department of Veterinary Pathobiology, Texas A&M University, College Station, TX, United States of America; 2 Department of Animal Genetics, Trakia University, Stara Zagora, Bulgaria; 3 Plum Island Animal Disease Center, U. S. Department of Homeland Security Science and Technology Directorate, Greenport, NY, United States of America; French National Centre for Scientific Research, FRANCE

## Abstract

African Swine Fever Virus (ASFV) is a high-consequence transboundary animal pathogen that often causes hemorrhagic disease in swine with a case fatality rate close to 100%. Lack of treatment or vaccine for the disease makes it imperative that safe and efficacious vaccines are developed to safeguard the swine industry. In this study, we evaluated the immunogenicity of seven adenovirus-vectored novel ASFV antigens, namely A151R, B119L, B602L, EP402RΔPRR, B438L, K205R and A104R. Immunization of commercial swine with a cocktail of the recombinant adenoviruses formulated in adjuvant primed strong ASFV antigen-specific IgG responses that underwent rapid recall upon boost. Notably, most vaccinees mounted robust IgG responses against all the antigens in the cocktail. Most importantly and relevant to vaccine development, the induced antibodies recognized viral proteins from Georgia 2007/1 ASFV-infected cells by IFA and by western blot analysis. The recombinant adenovirus cocktail also induced ASFV-specific IFN-γ-secreting cells that were recalled upon boosting. Evaluation of local and systemic effects of the recombinant adenovirus cocktail post-priming and post-boosting in the immunized animals showed that the immunogen was well tolerated and no serious negative effects were observed. Taken together, these outcomes showed that the adenovirus-vectored novel ASFV antigen cocktail was capable of safely inducing strong antibody and IFN-γ^+^ cell responses in commercial swine. The data will be used for selection of antigens for inclusion in a multi-antigen prototype vaccine to be evaluated for protective efficacy.

## Introduction

The African Swine Fever Virus (ASFV) is a high-consequence Transboundary Animal Disease (TAD) pathogen that causes hemorrhagic fever in swine and has mortality rates approaching 100% [1]. There is no vaccine or treatment available for this disease. The ASFV is a large enveloped double-stranded DNA icosahedral virus which exclusively infects the mammalian family of suids and argasid ticks of the genus *Ornithodoros*. This pathogen is responsible for major economic losses in endemic areas (sub-Saharan African countries and Sardinia) and poses a high risk to swine production in non-affected areas as it continues to spread globally [2]. Therefore, it is imperative that appropriate counter-measures are developed to reduce the prevalence of this disease in endemic areas, prevent further outbreaks in affected countries and safeguard the swine industries in non-affected areas.

Development of an efficacious vaccine for ASFV is still a challenge. There is strong evidence to suggest that protection against ASFV can be induced since attenuated virus has been shown to protect against parental or closely related virulent isolates [3–5]. Attenuated vaccines, however, are yet to be rigorously tested in the field in readiness for deployment. Development of an affordable DIVA (Differentiating Infected from Vaccinated Animals) ASFV subunit vaccine is a more attractive option, especially for use in non-endemic areas, in case of an outbreak.

Subunit vaccines based on one or two ASFV antigens have so far failed to induce immunity strong enough to confer significant protection among vaccinees [6–9], but, immunizing swine with DNA plasmids expressing a library of restriction enzyme digested ASFV-genome fragments conferred protection in a majority (60%) of the vaccinees against lethal challenge [10]. This result, though in favor of developing subunit based vaccines for ASFV, also highlights the main challenges associated with it, i.e. identification of protective antigens as well as a suitable delivery vector to induce strong protective responses. It is envisaged that successful development of an effective subunit vaccine will require empirical identification and validation of multiple suitable antigens that will induce significant protection in majority of the vaccinees.

We have previously shown that immunizing swine using a cocktail of replication deficient adenoviruses expressing ASFV antigens p32, p54, pp62 and p72 elicited robust antigen-specific antibody, IFN-γ^+^ cellular and cytotoxic T-lymphocyte (CTL) responses [11]. We used E1-deleted/replication-defective human adenovirus (Ad5) vector since it is safe, gives high protein expression levels and replicates at high titers in complementing cells making production scalable and reproducible [12, 13]. In addition, efficacy of adenoviruses in swine immunizations has previously been demonstrated in the successful development of a recently USDA-licensed recombinant Foot and Mouth Disease vaccine [14, 15]. In this study, we evaluated immunogenicity of seven ASFV vaccine candidates selected based on published literature (Table 1).

**Table 1:** Antigens selected for evaluation of immunogenicity.

Gene/Antigen	Functional Characteristics /Immune Relevance	Reference
A151R	Essential for the virus replication and morphogenesis. May play a role in viral transcription.	[16]
B119L	Critical for virus assembly. 90% of deletion mutants are crippled and fail to generate viable viral particles	[4], [17]
B602L	Chaperone for p72 (major capsid protein), repression leads to decrease of p72 expression, inhibition of pp220 and pp62 processing. Deletion severely alters viral assembly. Recognized by domestic pig and bush pig hyper-immune sera	[18], [19], [20], [21].
EP402R	ASFV Hemagglutinin. Extracellular domain contains protective T-cell epitopes. Protective immunity against homologous infection maybe haemadsorption inhibition (HAI) serotype-specific.	[22], [7], [23], [24]
B438L	Required for formation of vertices in icosahedral capsid	[25]
K205R	Induces strong antibody responses, but ability to elicit T-cell responses has not been tested. Recognized by domestic pig and bush pig hyper-immune sera	[20], [21]
A104R	Histone-like protein. Primes strong antibody responses, mainly detected in asymptomatic than chronically infected pigs. Presence of T-cell determinants has not been evaluated.	[26], [21]

The ability of these antigens to induce antibody and T-cell responses in commercial swine has not been evaluated so far. The antigen, EP402R, has been previously evaluated, however, only the extracellular domain was included and expressed as a fusion chimera along with other ASFV antigens, p32 and p54. In this study, we altered the EP402R protein sequence to delete the proline-rich repeats in the cytoplasmic tail and the resultant protein was designated EP402RΔPRR. The proline-rich repeats have been shown to interact with the adaptor protein SH3P7 in host cells and it is theorized that this interaction could, in part, be responsible for the immunomodulatory role of the EP402R protein [27]. Thus, deletion of the proline-rich repeats is expected to abrogate immunomodulatory effects when the EP402R protein is included in a multi-antigen subunit vaccine.

The focus of this work was to evaluate the immunogenicity of seven novel ASFV antigens, in commercial swine using replication-deficient adenovirus as a delivery platform with an end goal of identifying candidates for rationally designing a prototype multi-antigen ASFV subunit vaccine.

## Materials and methods

### Generation of recombinant adenoviruses expressing ASFV antigens

The amino acid sequences of the ASFV antigens (Georgia 2007/1 isolate) were obtained from Genbank (Accession FR682468). The EP402RΔPRR sequence was generated by deleting the proline-rich repeats from the EP402R cytoplasmic domain [27]. Since K205R and A104R polypeptides are short, they were fused in frame to generate a chimeric sequence, designated K205R-A104R. The coding sequences of the target antigens (A151R, B119L, B602L, EP402RΔPRR, B438L, and K205R-A104R) were then modified to add, in-frame, a FLAG- and HA- tag at the N- and C-termini, respectively, and the resultant amino acid sequences were used to generate synthetic genes which were codon-optimized for protein expression in swine. Synthesis, codon-optimization, cloning in pUC57 vector, and sequence-verification of these genes was outsourced (GenScript, NJ, USA). Each target gene was then amplified by PCR using *attB1*-FLAG specific forward and *attB2*-HA specific reverse primers and subcloned into Gateway pDonR221 vector (Invitrogen) as per manufacturer’s protocols. Positive pDonR clones were validated by sequencing and used to transfer the gene cassette into the adenovirus backbone vector, pAd/CMV/V5-DEST (Invitrogen) by homologous recombination. Validated positive pAd clones were then used to generate recombinant adenoviruses, designated AdA151R, AdB119L, AdB602L, AdEP402RΔPRR, AdB438L, and AdK205R-A104R, using the ViraPower Adenoviral Expression System (Invitrogen). Antigen expression by the adenoviruses was confirmed by immunocytochemistry of infected Human Embryonic Kidney (HEK)-293A cells (ATCC CRL-1573). One clone of each recombinant adenovirus was selected based on protein expression and amplified in a T75 tissue culture flask to make a working stock. The working stock was then used to infect up to 40 T175 flasks to generate bulk virus for immunization. The infected cells were harvested, lysed by freeze-thawing three times and the lysate was recovered. The titer of the virus in the cell lysates was determined by immunocytochemistry using QuickTiter Adenovirus Titer Immunoassay kit (Cell Biolabs, Inc.) protocol with a slight modification as previously described [11]. Briefly, HEK-293A cells infected with serial dilutions of the virus were first probed with rabbit anti-adenovirus polyclonal IgGs (1:500 dilution) (generated in-house), followed by an alkaline phosphatase-conjugated anti-rabbit IgG (1:1,000) (catalog number 711-055-152; Jackson Immuno-Research). FastRed TR/Naphthol AS-MX was then used as the substrate (F4523; Sigma). The stained cells were counted (20X objective) and the titer was calculated in IFU/ml. A recombinant adenovirus expressing luciferase, designated Ad-Luc, was similarly prepared and served as a negative control immunogen.

### Generation of recombinant ASFV antigens

The genes for antigens A151R, B602L, EP402RΔPRR, B438L, and K205R-A104R were PCR amplified from the respective pDonR clones using FLAG-specific forward and HA-specific reverse primers. The resultant PCR products were cloned into the pFastBac™ HBM TOPO shuttle vector (Invitrogen). Positive clones were identified by PCR screening, sequence-verified and used to generate recombinant baculoviruses using the Bac-to-Bac HBM TOPO Secreted Expression System (Invitrogen). Protein expression by the generated viruses was confirmed by immunocytochemistry of infected Sf-9 cells. One clone of each baculovirus was then scaled up and used to infect High Five cells (Invitrogen) to generate recombinant proteins. These proteins were affinity-purified using the anti-FLAG M2 affinity gel (Sigma, A2220). Recombinant B119L was affinity purified similarly, but from AdB119L-infected HEK-293A cell lysates.

### Validation of protein expression

#### Immunocytochemistry

Protein expression by the recombinant adenoviruses was evaluated by immunocytochemistry as described previously [28]. Briefly, HEK-293A cell monolayers infected with the recombinant adenoviruses, were probed with mouse anti-HA–alkaline phosphatase conjugate (Sigma, St. Louis, MO) diluted 1: 1,000 in Blocking buffer (PBS with 5% fetal bovine serum). Duplicate infected HEK-293A cell monolayers were first incubated with a gamma-irradiated convalescent swine serum (1:250 dilution)[11]. Following 3X washes, the cells were further incubated with a 1:500 dilution of alkaline phosphatase-conjugated goat anti-porcine IgG (Southern Biotech, Cat# 6050–04) for 1 hr. Following washes as above, Fast Red TR–Naphthol AS-MX substrate (Sigma, F4523) was added to the cells to detect the alkaline-phosphatase activity. Protein expression by the recombinant baculoviruses was similarly evaluated by infecting Sf-9 cells. Mock infected cells served as negative controls.

#### Western blot

Affinity-purified recombinant proteins (A151R, B119L, B602L, EP402RΔPRR, B438L, and K205R-A104R) were resolved by SDS-PAGE and transferred to Immobolin-P PVDF Membrane (Fisher Scientific). Following an overnight incubation at 4°C with blocking buffer (10% non-fat dry milk TBST), the membrane was incubated with ASFV-specific convalescent serum (1: 2,500 dilution in blocking buffer) for 1hr. The membrane was then washed 3X with TBST and incubated with peroxidase-conjugated Goat anti-swine IgG (1:5,000) (Jackson ImmunoResearch, Cat #114-035-003). Chemiluminescence was detected by the SuperSignal West Pico PLUS substrate (Thermo Scientific, Prod #34577).

#### Swine immunizations

Twenty weaned swine were randomly distributed into the treatment and control groups (n = 10). The treatment group was immunized with the Ad-ASFV cocktail (1 X 10^11^ IFU) of each construct (formulated in ENABL adjuvant (Benchmark Biolabs, Cat# 7010106-C6)). The control group received Ad-Luc (6 x 10^11^ IFU) formulated as above. The inoculum (5ml) was injected intramuscularly (1-2ml/site) in the neck area behind the ears. The animals were then boosted similarly after 8 weeks. Blood was collected for sera and PBMC isolation once pre-immunization and then biweekly post-prime, and then weekly post-boost for 3 weeks to run ELISAs and IFN-γ ELISPOTs. The animals were euthanized at 4 weeks post-boost.

### ELISA

Antigen-specific antibody responses were evaluated by an indirect ELISA as previously described [11]. Briefly, microplates coated overnight at 4°C with 100 μl of 1 μg/ml of affinity-purified antigen in bicarbonate coating buffer were washed and blocked with 10% non-fat dry milk in PBS with 0.1% Tween 20 for 1 hr. Sera were diluted at 1:100 (week 4 post-prime) or 1:8,000 (week 2 post-boost) in blocking buffer and added at 100 μl per well in triplicates. After incubation for 1 hr. at 37°C, the plates were washed and incubated for another hr. with 100 μl/well of a 1: 5,000 dilution of peroxidase-conjugated anti-swine IgG (Jackson ImmunoResearch, Cat# 114-035-003). Following washes, the plates were developed with Sure Blue Reserve TMB substrate (KPL, Cat# 53-00-02) and reaction was stopped using 1N Hydrochloric acid. The absorbance at 450 nm was read using BioTek microplate reader (Synergy H1 Multi-mode reader). The IgG response by each animal to each antigen was calculated as mean absorbance of test sera minus the mean absorbance of the cognate pre-immunization sera. To determine antigen-specific IgG end-point titers, sera from blood collected two weeks post-boost was serially diluted two-fold starting at 1: 4,000 up to 1: 4 X 10^6^. The pre-immunization serum was similarly diluted. The end-point titer was calculated as described previously [11].

### Indirect Fluorescence Antibody assay (IFA)

Pretreated Teflon coated slides with fixed ASFV (Georgia 2007/1)-infected and mock-infected VERO cells (ATCC CCL-81) were used to perform the IFA as previously described [11]. Briefly, the slides were incubated with sera from two weeks post-boost diluted 1:250 for 1 hr. at 37°C. ASFV-specific convalescent serum (1: 10,000) was used as a positive control and normal swine serum (1:250) (GIBCO) was used as a negative control. Following extensive washes with D-PBS, the wells were incubated with FITC-conjugated goat anti-swine sera (Kirkegaard and Perry Cat No. 02-14-02) for 45 minutes at 37°C, washed again and mounted with Prolong Gold antifade reagent with DAPI (Invitrogen, Cat. No. PT389868). The cells were visualized at a 40X magnification using an Olympus immuno-fluorescent microscope (model BX-40) and photographed by an Olympus digital camera (model DP 70). The IFAs were conducted at Plum Island Animal Disease Center.

### Western blot with ASFV-infected cell lysates

Lysates from ASFV Georgia 2007/1 (VERO cell-adapted)-infected VERO cells were used to perform a western blot as previously described [11]. Briefly, the prepared cell lysates were electrophoresed on a NuPAGE 4–12% Bis-Tris Gel (1.0mm X 2D well) for 35 mins, followed by transfer to 0.2 μm PVDF membranes (Invitrogen #LC2002) for 1 hour. The membranes were then blocked for 1 hr. in blocking buffer (PBST+5% non-fat dry milk) and transferred to the Protean II Slot-Blotter. Sera from week 2 post-boost were diluted 1:250 in blocking buffer and added to individual wells for 1 hr. at room temperature with shaking. After washing the wells 3X with PBST, the membranes were removed from the blotting apparatus and incubated for 1 hr. with a 1: 2,000 dilution of Goat anti-swine-HRP (KPL #14-14-06). Following washes, the membranes were developed using DAB (Sigma #D4293). ASFV-specific convalescent serum (1:10,000) was used as a positive control and normal swine serum (1:200) was used as a negative control. Background reactivity to host-cell antigens was gauged similarly using mock-infected lysates. The western blot analysis was carried out at Plum Island Animal Disease Center.

### IFN-γ ELISPOT assays

Antigen-specific IFN-γ^+^ cell response was evaluated by an enzyme-linked immunospot (ELISPOT) assay using the Mabtech kit (Cat# 3130-2A), as per manufacturer’s instructions and as previously described [11]. Briefly, whole blood-derived PBMCs resuspended in complete RPMI-1640 media were added to wells of MultiScreen-HA plates (Millipore) at a density of 250,000 cells/well. Affinity-purified antigens were added to the cells at a final concentration of 2.5 μg/ml in triplicates. Phytohemagglutinin (PHA) mitogen (5 μg/ml) was used as a positive control, whereas media served as the negative control. The spots were counted by an ELISPOT reader and AID software (AutoImmun Diagnostica V3.4, Strassberg, Germany). The mean number of IFN-γ^+^ Spot-Forming Cells (SFC) for each sample was calculated by subtracting the mean number of spots in the negative control wells from the mean number of spots in the sample wells. The data is presented as mean number of SFC per 10^6^ PBMCs.

### Statistical analysis

The differences in the mean antigen-specific antibody and IFN-γ^+^ responses between the treatment and the control group were analyzed by an unpaired t-test with Welch’s correction, and a *P* value of ≤0.05 was considered significant. The analysis was performed with GraphPad Prism Version 6.05 using a significance level of P<0.05.

### Ethics statement

All animal procedures were conducted as per the Animal Use Protocol 2014–0020, reviewed and approved by the Texas A&M University Institutional Animal Care and Use Committee (IACUC). The Texas A&M IACUC follows the regulations, policies and guidelines outlined in the Animal Welfare Act (AWA), USDA Animal Care Resource Guide and the PHS Policy on Humane Care and Use of Laboratory Animals. The animals were monitored twice daily for any clinical signs and to document any localized and or systemic adverse effects. At the termination of the study, the animals were euthanized with an overdose of sodium pentobarbital.

## Results and discussion

### Recombinant constructs encoding ASFV antigens

**Fig 1:**
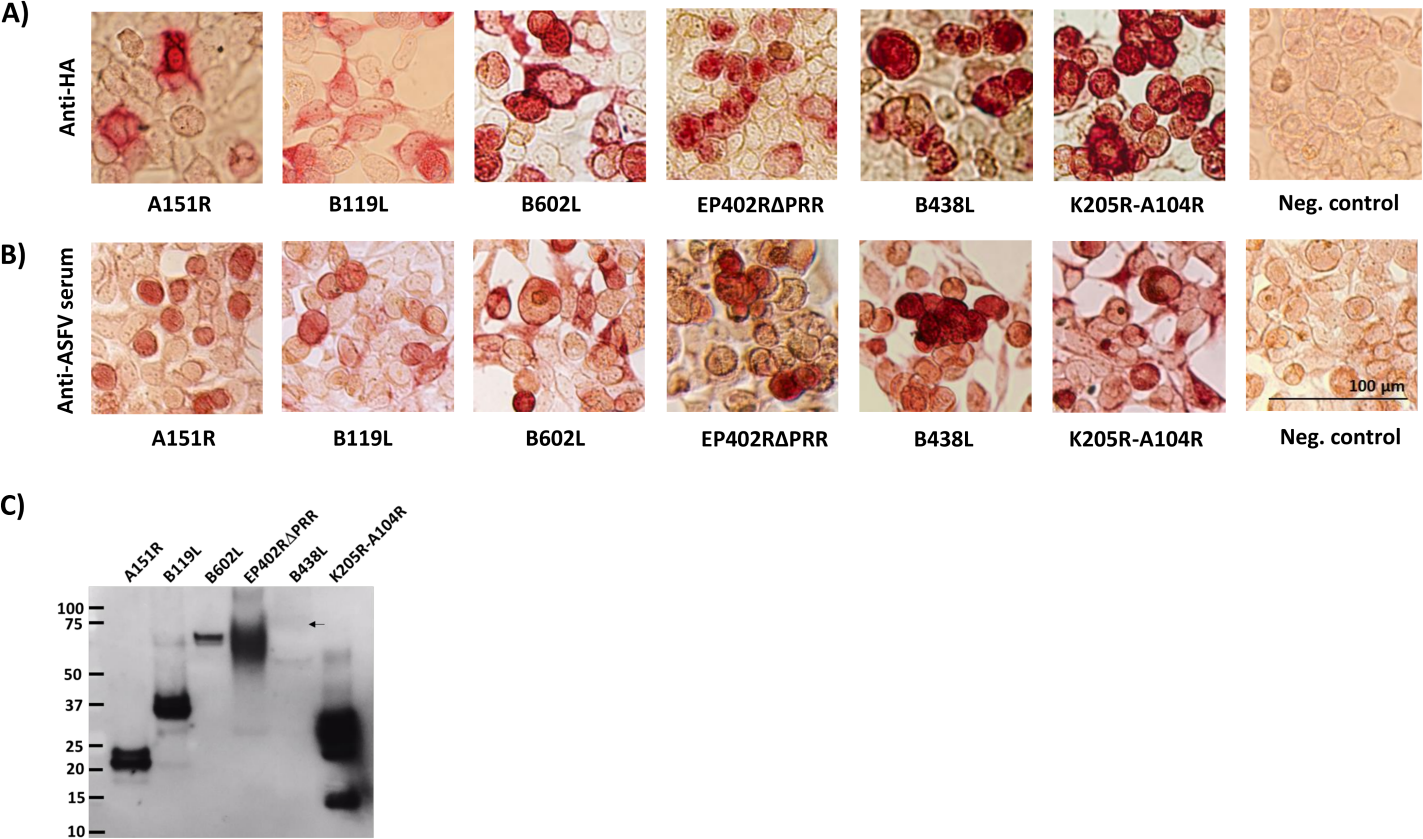
Protein expression by ASFV constructs. The expression and authenticity of the ASFV antigens encoded by the generated recombinant constructs was evaluated by immunocytochemistry and western blot analysis. Panels: A) HEK-293A cells infected with recombinant adenoviruses and probed with anti-HA mAb; and B) HEK-293A cells infected with recombinant adenoviruses and probed with gamma-irradiated ASFV-specific convalescent serum. Negative controls are mock infected HEK-293A cells. C) A western blot of the affinity purified ASFV proteins probed with the convalescent serum. The molecular weights are expressed in kDa. The arrow points to the faint band detected for B438L (the signal intensity of the band increased with longer exposure times).

Codon-optimized synthetic genes encoding antigens, A151R, B119L, B602L, EP402RΔPRR, B438L, and K205R-A104R fused in-frame to FLAG and HA tags were used to generate recombinant adenoviruses designated AdA151R, AdB119L, AdB602L, AdEP402RΔPRR, AdB438L, and AdK205R-A104R. The immunogenicity of K205R and A104R was evaluated as a chimera since both proteins are relatively small (~20kDa and ~10kDa) and delivering them *in vivo* as a chimera would reduce the number of adenoviruses to be inoculated. Evaluation of protein expression by immunocytochemistry of adenovirus-infected HEK-293A cells using anti-HA mAb and the ASFV-specific convalescent serum showed that the assembled recombinant adenoviruses expressed full-length authentic ASFV antigens (Fig 1A and 1B). The synthetic ASFV genes were also used to generate recombinant baculoviruses for generation of affinity-purified recombinant proteins needed for *in vitro* evaluation of antigen-specific antibody and cell responses. However, despite several attempts, we were unsuccessful in generating a recombinant baculovirus expressing B119L and thus we used affinity-purified antigen from AdB119L-infected HEK-293A cells for *in-vitro* readouts. The authenticity of the affinity-purified recombinant proteins was validated by western blot using ASF-specific convalescent serum (Fig 1C). Strong bands were detected for all antigens except B438L. The predicted molecular weight of B438L (with the FLAG and HA tags) is ~56kDa. A very faint diffused band (depicted by an arrow) slightly lower than 75 kDa was observed for antigen B438L. The antigen loads had been optimized for signal detection, however, for antigen B438L a strong signal was not detected despite increasing antigen load to microgram quantities (see S1 Fig). This could be a result of low B438L-specific antibodies in the ASFV-specific convalescent serum. To confirm this observation, we performed western blots of antigens A151R (as a positive control) and B438L, probed with either an anti-HA mAb or the ASFV-specific convalescent serum (S2 Fig). The band at slightly <75kDa was detected with the anti-HA mAb for antigen B438L, however, no signal was seen with the convalescent serum. This validates that the absence of a strong signal for B438L in the western blot in Fig 1C was indeed due to low B438L-specific antibodies (also confirmed by an ELISA discussed later in the manuscript) in the serum and not an insufficient antigen load.

### Ad5-ASFV cocktail primed ASFV antigen-specific antibodies

**Fig 2:**
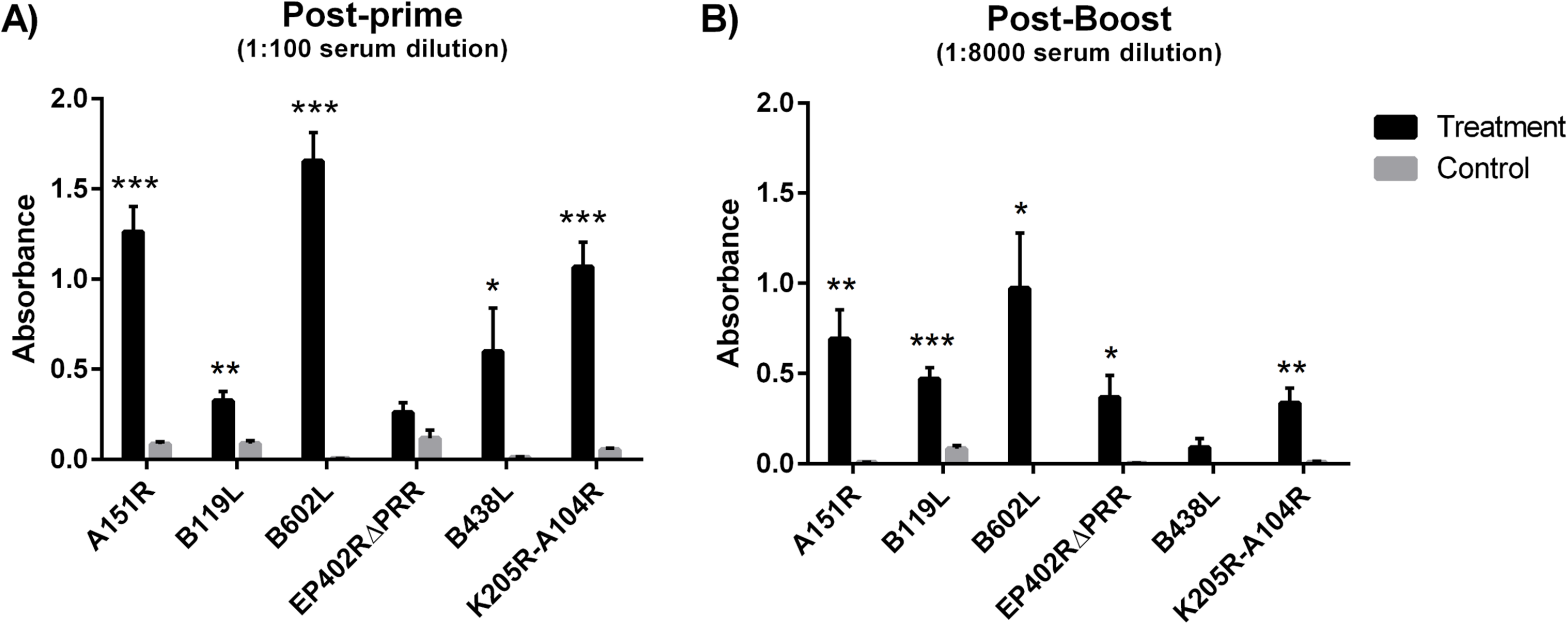
Mean antigen-specific IgG responses post-priming and post-boost. Antigen-specific IgG response was evaluated post-prime and post-boost by ELISA. A) Sera from week 4 post-prime were evaluated at a 1:100 dilution. B) Sera from week 2 post-boost were evaluated at a 1:8,000 dilution (to prevent the absorbance values from going out of range). The error bars represent the SEM. The asterisks denote a significant difference between the mean response of the treatment and control animals. *p<0.05, **p<0.01, ***p<0.001.

**Fig 3:**
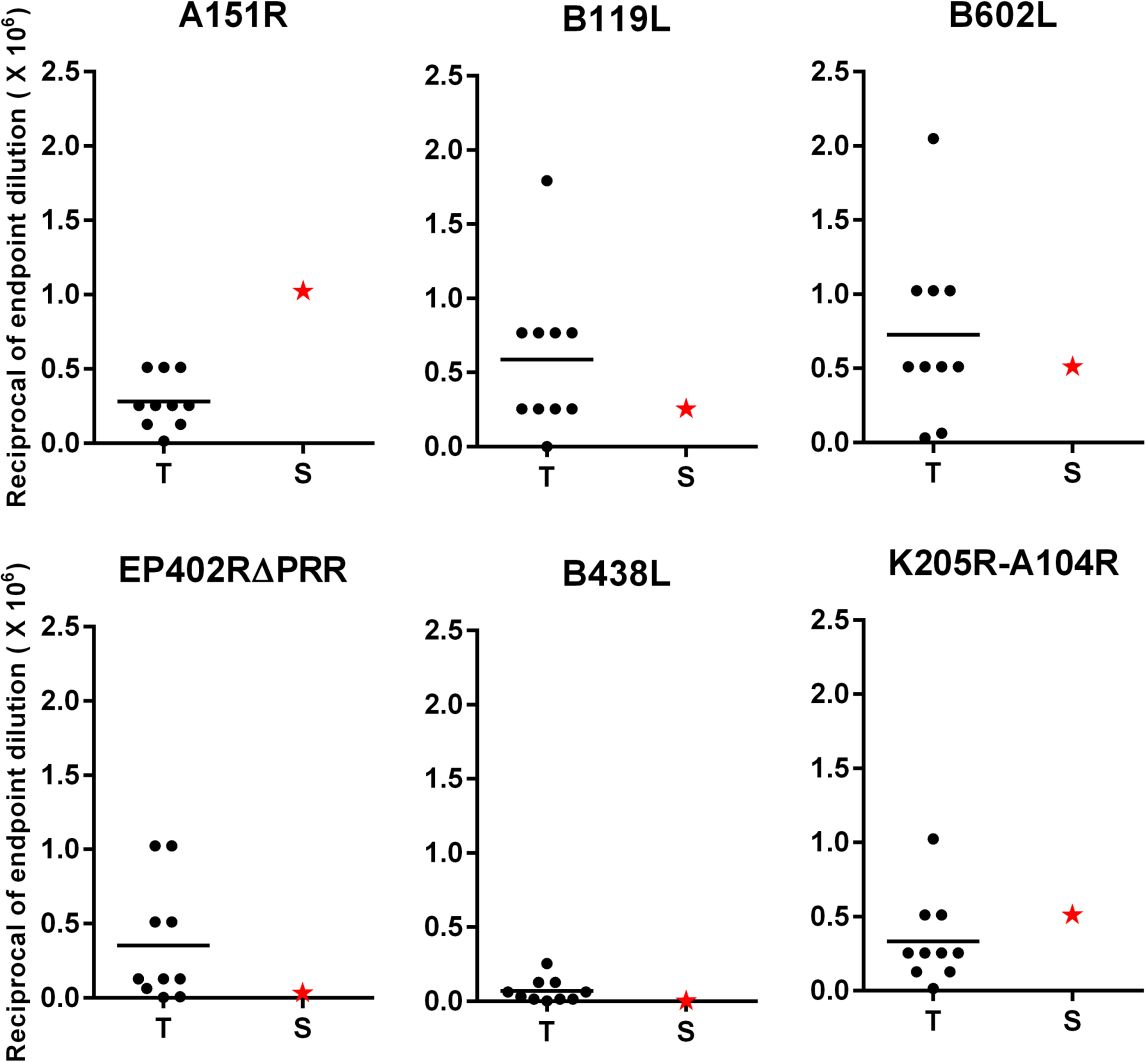
Antigen-specific end-point IgG titers. Antigen specific antibody titers, determined by ELISA, in sera from treatment group animals (T) collected two weeks post-boost. The dilution of the sera at which the absorbance reading was higher than that of the cognate pre-bleed +3 standard deviations is reported as the end-point titer. The ASFV-specific convalescent serum was titrated similarly and is represented by the red star symbol (S). Data is represented as the reciprocal of the end-point sera dilution x 10^6^. For antigen, B119L the sera from control group animals was also titrated to gauge background reactivity to host-cell and vector-derived antigens. An average of the titers of the control group animals was then subtracted from the titer of each treatment group animal to give B119L-specific titers. For the remaining antigens, the post-boost sera from the control group animals showed no reactivity as seen in Fig 2B.

Twenty commercial swine were randomly divided into two groups (n = 10). Animals of the treatment group were immunized with a cocktail of six recombinant adenoviruses expressing the A151R, B119L, B602L, EP402RΔPRR, B438L, and K205R-A104R ASFV antigens, whereas the negative control group received Ad-Luc sham treatment. After priming, antigen-specific IgG responses were detected in a majority of swine in the treatment group, but not the control group. Data from sera analyzed four weeks post-priming is shown (Fig 2A). The mean response of the treatment group was significantly higher than the control group for antigens A151R (p<0.001), B119L (p<0.01), B602L (p<0.001), B438L (p<0.05) and K205R-A104R (p<0.001). The mean antibody response against the EP402RΔPRR antigen by the swine in the treatment group was slightly higher than the controls but not significant. The strong mean responses observed against antigens B602L and K205R-A104R is consistent with previous studies where these antigens have been shown to be strongly recognized by domestic pig and bush pig hyper-immune sera [20, 21]. Following boosting at 8 weeks post-priming, antigen-specific recall IgG responses against all antigens were detected in the animals in the treatment group (Fig 2B). The mean response of the treatment group was significantly higher than the control group for antigens A151R (p<0.01), B119L (p<0.001), B602L (p<0.05), EP402RΔPRR (p<0.05), and K205R-A104R (p<0.01), but not for antigen B438L. It is important to note that the responses at week 2 post-boost were evaluated at 1:8,000 sera dilution, whereas the responses post-prime were evaluated at a 1:100 sera dilution (Fig 2). This eliminated the background responses observed against some antigens post-prime in the control group. However, for antigen B119L, the control group still had a low-level of background reactivity after boosting. This background response could be attributed to vector and host-cell line (HEK-293A)-specific antibodies since the affinity-purified B119L antigen was derived from lysates of AdB119L-infected HEK-293A cells. Also the response seen in the treatment group is also likely to be inclusive of a low level of vector and host-cell line specific antibodies. Evaluation of antigen-specific end-point titers post-boost in the immunized pigs showed that a majority of the vaccinees had titers ≥ 1:256 x 10^3^ against antigens A151R, B119L, B602L, and K205R-A104R (Fig 3). The highest titer was 1:2 x 10^6^ against B602L in one of the vaccinees (Fig 3). A comparison of the antigen-specific titers in sera from the vaccinees with the titer of the ASFV-specific convalescent serum revealed that Ad-ASFV cocktail was able to induce titers higher or equivalent to the convalescent serum in a majority of animals for antigens B119L (90% of vaccinees), B438L (90% of vaccinees), B602L (80% of vaccinees), and EP402RΔPRR (80% of vaccinees). This is a noteworthy result, since these animals received only two immunizations of the Ad-ASFV cocktail, whereas the positive control convalescent serum came from an animal that received multiple inoculations of live ASFV [11]. However, for antigen, K205R-A104R only 3 of 10 vaccinees had titers that matched up to the convalescent serum, whereas for antigen, A151R the titers induced in the vaccinees did not match up to the convalescent serum.

The role of antibodies in ASFV protection is not yet completely understood, however strong evidence in favor of antibodies (reviewed in [29]) and importantly, protection reported by passively acquired anti-ASFV antibodies supports the evaluation of humoral responses in immunogenicity studies focused on identification of novel targets for subunit vaccine development [30, 31]. In the current study, a cocktail of replication-incompetent adenovirus constructs expressing multiple ASFV antigens primed strong antibody responses against all antigens in a majority of the animals.

### Antibodies triggered by the Ad5-ASFV cocktail recognized ASF virus

**Fig 4:**
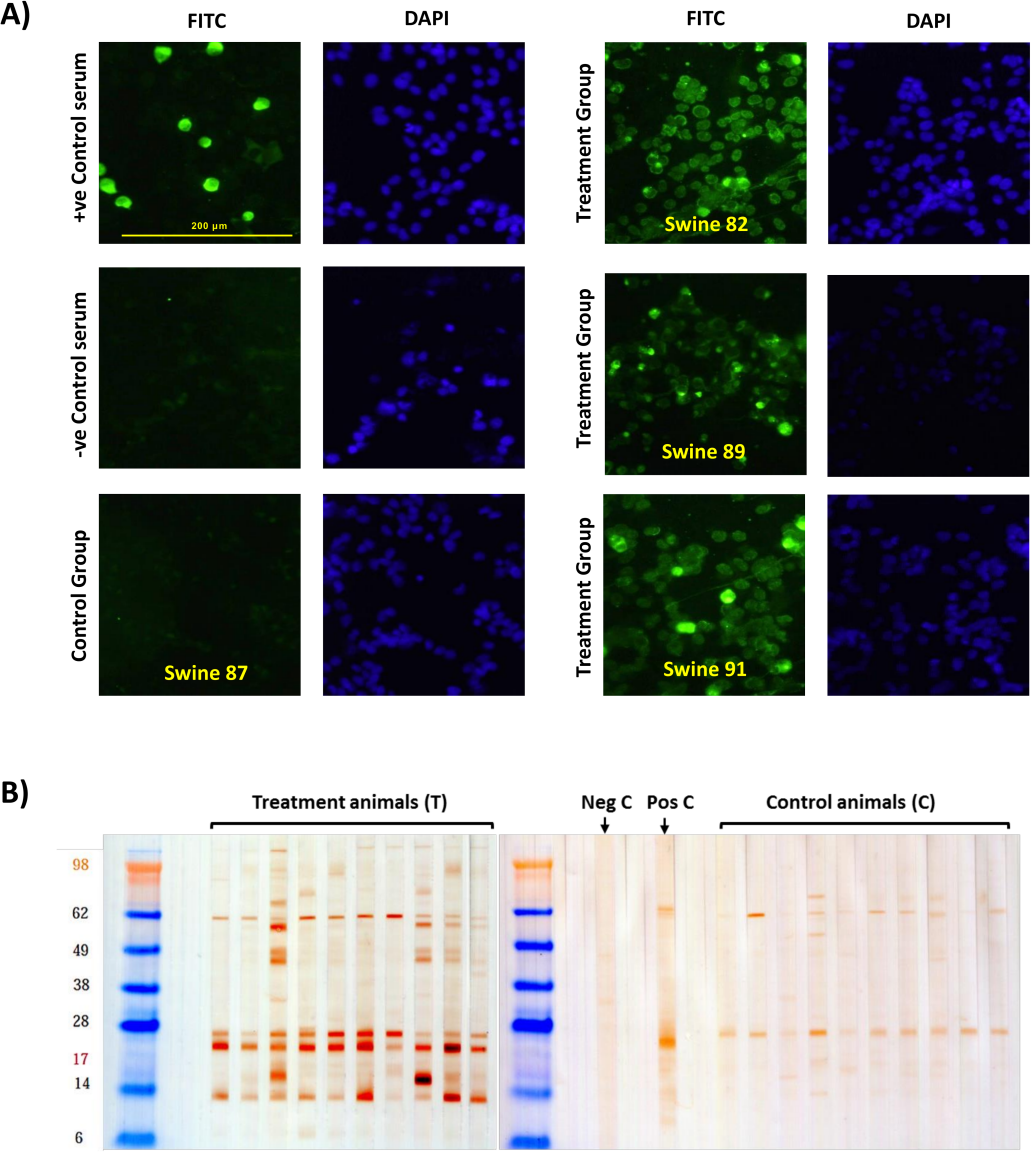
Antibodies primed by the Ad-ASFV cocktail recognized ASF virus. Analysis of sera from two weeks post-boost by Indirect Fluorescence Antibody assay (IFA) and western blot showed that the antibodies primed by the Ad-ASFV cocktail recognized the parental ASFV infected cells and ASFV-derived antigens. Panel A) Vero cells infected with ASFV Georgia 2007/1 probed with sera from treatment and negative control animals. ASFV specific convalescent serum was used as the positive control and normal swine serum served as the negative control. Data for three animals (that gave the strongest reaction) from the treatment group and one animal from the control group is shown. A summary of IFA results for all animals is presented in Table 2. B) Lysates from Vero cells infected with ASFV Georgia 2007/1 isolate were blotted and probed with sera from all animals. Normal swine serum was the negative control and ASFV-specific convalescent serum was the positive control. Differences in coloration are due to actual band intensities; darker color is higher concentration of antibody bound to antigen (antigen concentration is constant).

Indirect Immunofluorescence Antibody Assay (IFA) performed with sera from blood collected from the vaccinees two weeks post-boost, confirmed that the antibodies triggered by the Ad5-ASFV cocktail recognized VERO cells infected with the actual ASF virus (Georgia 2007/1 isolate) but not mock-infected cells (Fig 4A). Sera from 8 out of 10 swine in the treatment group, but none from the controls, recognized the ASFV-infected cells (Table 2). Sera from 2 animals (swine 89 and swine 91) were most reactive and reacted with the plasma membrane, a virus–factory like structure and general cytoplasm. Western blot analysis of ASFV-infected Vero cell lysates probed with the post-boost sera also validated the above results (Fig 4B). This outcome showed that synthetic genes encoding antigens of ASFV (a Risk Group 3 pathogen) that requires BSL3 biocontainment can safely be used at BSL2 level to develop and test immunogenicity and tolerability of prototype ASFV vaccines. These results, however do not directly demonstrate that the ASFV-specific antibodies have functional activity. In case of ASFV, it is generally acceptable in the scientific community, that conventional plaque reduction assay to measure ASFV antibody neutralization activity is technically difficult since low-passage (virulent) ASFV strains show no or a significant delay in plaque formation, and is especially difficult to conduct the assay in primary swine macrophage cells. A highly attenuated ASFV Georgia strain that is adapted to a suitable cell line (e.g., VERO cells), or a genetically modified ASF virus expressing a chromogenic marker gene, for use in testing study samples for virus neutralization activity was not available at the time the study was conducted.

**Table 2:** Summary of IFA results.

Treatment Group: Swine No.	Reactivity		Control Group: Swine No.	Reactivity	
	ASFV-infected Vero cells	Mock-infected Vero cells		ASFV-infected Vero cells	Mock-infected Vero cells
76	+++	-	77	-	-
78	-	-	79	-	-
81	++	-	80	-	-
82	+++	-	84	-	-
83	-	-	85	-	-
89	+++	-	87	-	-
90	+	-	88	-	-
91	++++	-	93	-	-
92	+	-	95	-	-
96	+++	-	99	-	-
ASFV convalescent serum	++++	-	Normal serum	-	-

The number of ‘+’ signs represents the comparison between the intensity of a positive signal from the sera of the animals and that from the ASFV-specific convalescent serum (positive control).

‘++++’: signal as strong as positive control

‘+’: weakest but positive signal

‘-‘: No signal detected

### Ad5-ASFV cocktail primed IFN-γ-secreting cells

**Fig 5:**
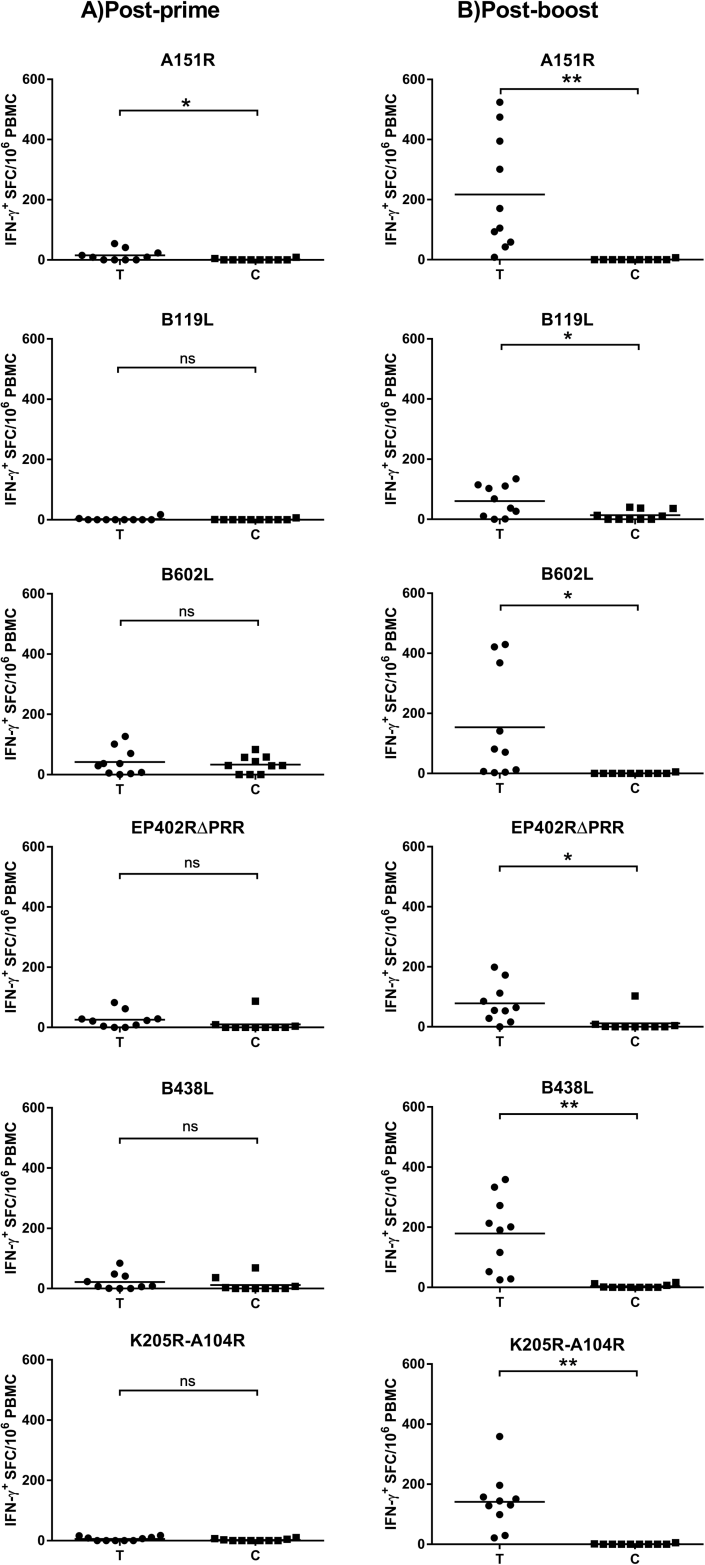
ASFV antigen-specific IFN-γ response post-prime and post-boost. The frequency of antigen-specific IFN-γ-secreting cells in PBMCs collected post-prime and post-boost was evaluated by IFN-γ ELISPOT assays. Data for A) One week post-prime; and B) three weeks post-boost is shown. The response is presented as IFN-γ Spot Forming Cells (SFC)/106 PBMCs. The mean response of the treatment group (T) was compared to the mean response of the control group (C) using an unpaired t-test with Welch’s correction. ** represents p<0.01, * represents p<0.05 and ‘ns’ stands for a non-significant difference.

Low frequencies of antigen-specific IFN-γ responses were detected in a few animals by IFN-γ ELISPOT analysis of PBMCs collected one-week post-priming (Fig 5A). Specifically, a significant difference (p<0.05) between the mean response of the treatment group and negative control group animals was detected only for antigen A151R (Fig 5A). However, after boosting, strong recall IFN-γ^+^ responses were detected in a majority of animals for all the antigens (Fig 5B). The mean response of the treatment group was significantly higher than the control group for all antigens (p<0.05 for antigens B119L, B602L, EP402RΔPRR, and p<0.01 for antigens A151R, B438L, and K205R-A104R). The IFN-γ ELISPOT data clearly showed that the homologous booster dose was able to sufficiently amplify the primary response to give strong recall responses against all antigens in a majority of the vaccinees (Fig 5). The high frequencies of antigen-specific IFN-γ^+^ cellular responses induced are promising in light of the results reported from other subunit vaccine studies. Notably, immunization with an ubiquitin tagged chimera of antigens p30, p54 and CD2v using DNA plasmids conferred protection against lethal challenge in some of the vaccinees [7]. In addition, in another study, by the same authors, immunizing animals with BacMams expressing the same antigen chimera (p30, p54 and CD2V) conferred partial protection upon a sub-lethal challenge, and a direct correlation between protection and ASFV-specific IFN-γ^+^ response was observed [6]. Interestingly, in both studies the IFN-γ response against the extracellular domain of EP402R was negligible. We have shown that the adenovirus-vectored EP402RΔPRR induced strong antigen-specific IFN-γ^+^ responses in 70% of the vaccinees post-boost.

### Ad5-ASFV cocktail was well tolerated

Following inoculation of the Ad-ASFV cocktail, three swine in the treatment group were observed to be depressed and one had mild fever on the first day. However, all the swine were normal on all subsequent days. After boosting, one pig in the treatment group was observed to be depressed and had fever that required treatment with Banamine. All the swine in the negative control group were normal post-priming and post-boosting. Overall, the Ad-ASFV cocktail was well tolerated with no adverse effects.

The overall outcome is evidence that a vaccine formulated using a cocktail of replication-incompetent adenovirus expressing protective ASFV antigens is likely to be well tolerated by commercial swine at doses as high as 10^11^ IFU used in a homologous prime-boost immunization regimen. This scenario is anticipated since effective ASFV subunit vaccines will likely require delivery of multiple antigens given that studies conducted so far have shown that a combination of one or a few antigens does not confer complete protection.

## Conclusion

The African Swine Fever Virus (ASFV) continues to pose a high risk to the swine industry and it is still causing economic losses in endemic areas. Since there is no vaccine or treatment available yet, it is important to identify viral proteins that can elicit strong immune responses and therefore be considered viable candidates for subunit vaccine development. We have optimized an adenovirus-vector based ASFV antigen delivery system which allows for immunization of swine with multiple ASFV antigens and the subsequent evaluation of their immunogenicity. The robust antigen-specific IFN-γ^+^ responses induced by the adenovirus vector against all the antigens tested in this study as well as other ASFV antigens evaluated in our previous study make it a promising delivery platform for testing vaccine candidates for protection against ASFV [11]. Upon investigation of antigen-specific responses of individual animals, we observed a significant (p<0.05) positive correlation between the antigen-specific IFN-γ response and the antigen-specific end-point antibody titers for 4 of the 6 antigens (see S3 Fig). An interesting observation, in regards to antigen B438L, is the relatively low humoral responses in contrast to the strong IFN-γ^+^ responses induced (Fig 3, Fig 5, S3 Fig). The inability of this antigen to induce strong antibody responses was corroborated by the fact that the ASFV-specific convalescent serum also had a comparatively low B438L-specific titer (1:4,000). Thus, even though B438L does not induce a high antibody response, it still is an attractive candidate for future efficacy studies based on its ability to induce strong IFN-γ^+^ cell responses. This study also showed that an adenovirus-based ASFV vaccine can be used successfully for homologous prime-boost vaccination. If this approach is shown to confer protection, it will cut costs incurred by use of a heterologous prime-boost immunization strategy. Thus, these findings support use of the replication-incompetent adenovirus as a vector for the development of a commercial vaccine for protection of pigs against African swine fever virus. The next logical step is to test whether these multiple ASFV antigens delivered in a cocktail format can confer protection in a challenge study.

## Supporting information

S1 Fig SDS-PAGE of affinity purified ASFV antigens. Coomassie (Thermo Scientific Imperial Protein Stain) stained gel of affinity-purified recombinant ASFV proteins. The protein load for each of the antigens was 1μg based on BCA assay. The affinity-purified preps for antigens B119L and B438L contain other contaminating proteins. For antigen B119L, the band detected on the western (~40 kDa) (Fig 1C) is faint but visible on the stained gel. For antigen B438L, the arrow points to the faint band detected on the western blot (Fig 1C). The amount of sample loaded for the western blot was 0.1 to 1X the amount on the stained gel (to achieve optimal band intensity when probed with the convalescent sera) for all antigens except B438L. For antigen B438L, the amount loaded on the western blot (in Fig 1C) was increased to 8X (8 μg) the amount on the stained gel, to enable detection of the faint band.
(PDF)

S2 Fig SDS PAGE and western blots of antigen B438L. A) Coomassie (Thermo Scientific Imperial Protein Stain) stained gel of affinity-purified recombinant ASFV proteins, A151R (used as a control) and B438L; B) Western blot of proteins A151R and B438L probed with anti-HA mAb; and C) Duplicate western blot probed with ASFV-specific convalescent serum. The protein load for both antigens on the western blots is 0.1X the load on the PAGE.
(PDF)

S3 Fig Antigen-specific correlation between IFN-γ and antibody response. Correlation analysis of antigen-specific IFN-γ and antibody titers of individual animals revealed a significant (p<0.05) positive correlation for all antigens except EP402RΔPRR and B438L. The Pearson correlation coefficient (r) and the statistical significance for each correlation is shown. *** represents p<0.001, ** represents p<0.01, * represents p<0.05 and ‘ns’ stands for a non-significant difference.
(PDF)
