# Supplementary material for: Adenovirus-vectored novel African Swine Fever Virus antigens elicit robust immune responses in swine
Source: PLoS One. 2017 May 8;12(5):e0177007. doi: 10.1371/journal.pone.0177007 (PMC5421782; doi:10.1371/journal.pone.0177007)
Supplement: S1 Fig — Coomassie (Thermo Scientific Imperial Protein Stain) stained gel of affinity-purified recombinant ASFV proteins. The protein load for each of the antigens was 1μg based on BCA assay. The affinity-purified preps for antigens B119L and B438L contain other contaminating proteins. For antigen B119L, the band detected on the western (~40 kDa) (Fig 1C) is faint but visible on the stained gel. For antigen B438L, the arrow points to the faint band detected on the western blot (Fig 1C). The amount of sample loaded for the western blot was 0.1 to 1X the amount on the stained gel (to achieve optimal band intensity when probed with the convalescent sera) for all antigens except B438L. For antigen B438L, the amount loaded on the western blot (in Fig 1C) was increased to 8X (8 μg) the amount on the stained gel, to enable detection of the faint band. (PDF) [file pone.0177007.s001.pdf]

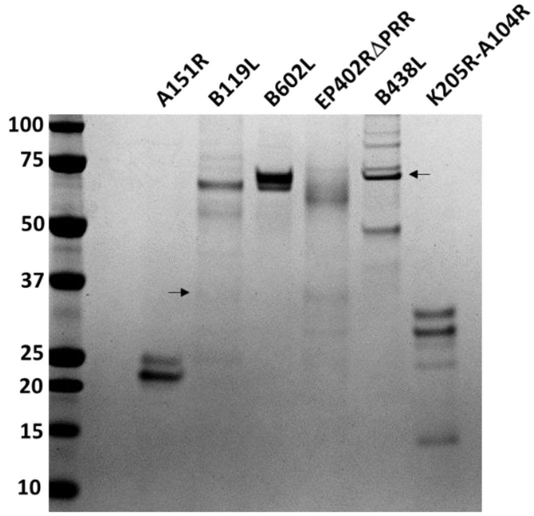

S1 Fig. SDS-PAGE of affinity purified ASFV antigens

Coomassie (Thermo Scientific Imperial Protein Stain) stained gel of affinity-purified recombinant ASFV proteins. The protein load for each of the antigens was 1µg based on BCA assay. The affinity-purified preps for antigens B119L and B438L contain other contaminating proteins. For antigen B119L, the band detected on the western (~40 kDa) (Fig. 1C) is faint but visible on the stained gel. For antigen B438L, the arrow points to the faint band detected on the western blot (Fig. 1C). The amount of sample loaded for the western blot was 0.1 to 1X the amount on the stained gel (to achieve optimal band intensity when probed with the convalescent sera) for all antigens except B438L. For antigen B438L, the amount loaded on the western blot (in Fig. 1C) was increased to 8X (8 µg) the amount on the stained gel, to enable detection of the faint band.
