# Supplementary material for: Adenovirus-vectored novel African Swine Fever Virus antigens elicit robust immune responses in swine
Source: PLoS One. 2017 May 8;12(5):e0177007. doi: 10.1371/journal.pone.0177007 (PMC5421782; doi:10.1371/journal.pone.0177007)
Supplement: S2 Fig — A) Coomassie (Thermo Scientific Imperial Protein Stain) stained gel of affinity-purified recombinant ASFV proteins, A151R (used as a control) and B438L; B) Western blot of proteins A151R and B438L probed with anti-HA mAb; and C) Duplicate western blot probed with ASFV-specific convalescent serum. The protein load for both antigens on the western blots is 0.1X the load on the PAGE. (PDF) [file pone.0177007.s002.pdf]

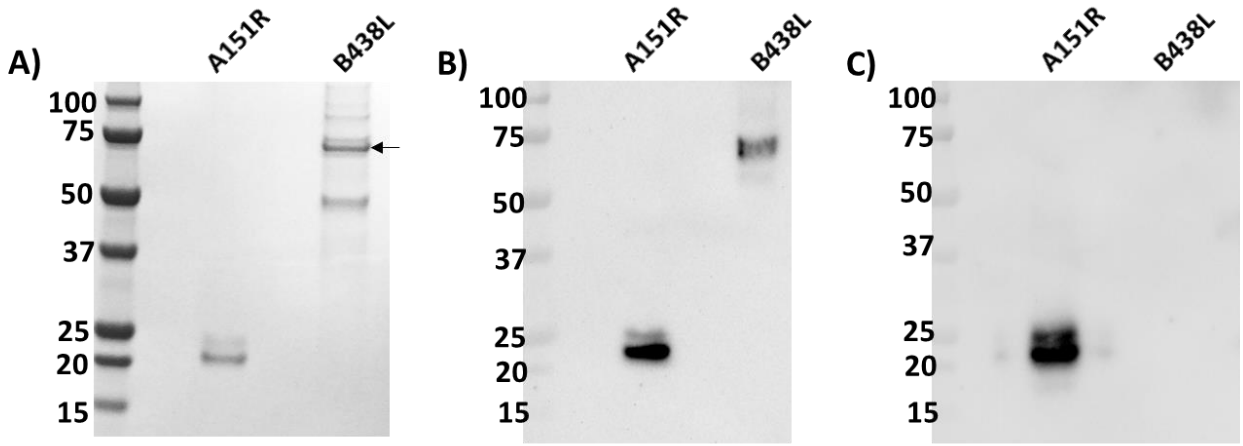

S2 Fig. SDS PAGE and western blots of antigen B438L

A) Coomassie (Thermo Scientific Imperial Protein Stain) stained gel of affinity-purified recombinant ASFV proteins, A151R (used as a control) and B438L. B) Western blot of proteins A151R and B438L probed with anti-HA mAb. C) Duplicate western blot probed with ASFV-specific convalescent serum. The protein load for both antigens on the western blots is 0.1X the load on the PAGE.
