# Supplementary material for: Adenovirus-vectored novel African Swine Fever Virus antigens elicit robust immune responses in swine
Source: PLoS One. 2017 May 8;12(5):e0177007. doi: 10.1371/journal.pone.0177007 (PMC5421782; doi:10.1371/journal.pone.0177007)
Supplement: S3 Fig — Correlation analysis of antigen-specific IFN-γ and antibody titers of individual animals revealed a significant (p<0.05) positive correlation for all antigens except EP402RΔPRR and B438L. The Pearson correlation coefficient (r) and the statistical significance for each correlation is shown. *** represents p<0.001, ** represents p<0.01, * represents p<0.05 and ‘ns’ stands for a non-significant difference. (PDF) [file pone.0177007.s003.pdf]

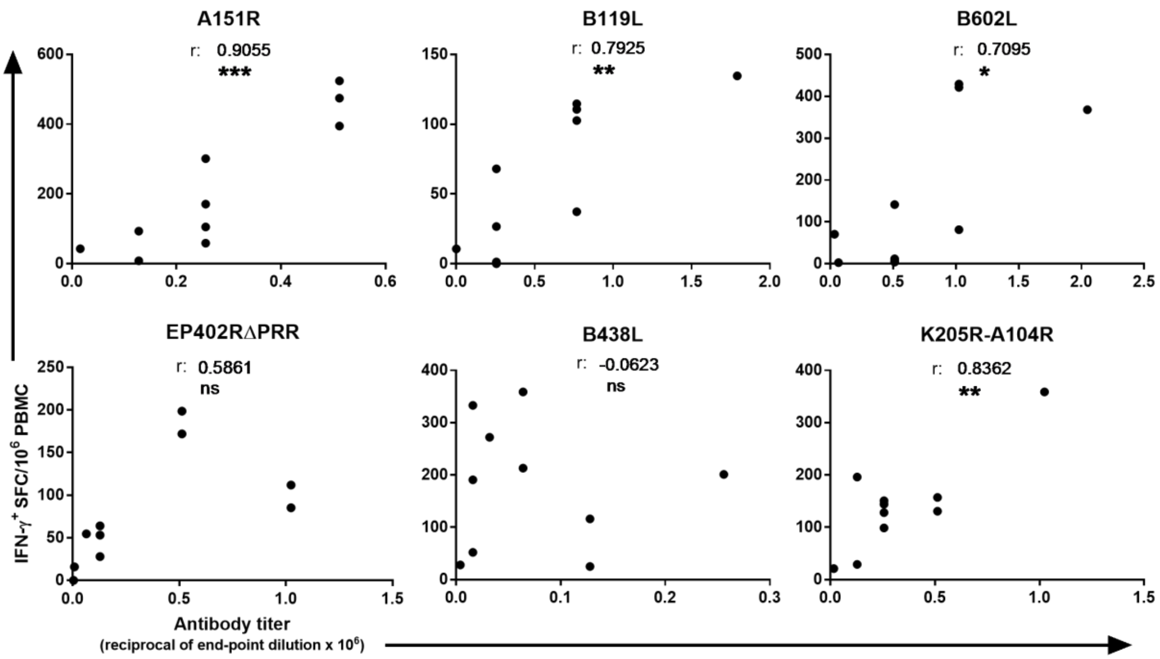

S3 Fig. Antigen-specific correlation between IFN-γ and antibody response.

Correlation analysis of antigen-specific IFN-γ and antibody titers of individual animals revealed a significant ($p<0.05$) positive correlation for all antigens except EP402RΔPRR and B438L. The Pearson correlation coefficient (r) and the statistical significance for each correlation is shown. *** represents $p<0.001$, ** represents $p<0.01$, * represents $p<0.05$ and 'ns' stands for a non-significant difference.
